# Plasmonic photothermal printing of all-metal-oxide electronics

**DOI:** 10.1038/s41377-025-01964-1

**Published:** 2025-09-15

**Authors:** Eric Mazur

**Affiliations:** School of Engineering and Applied Sciences, Harvard University, Cambridge, Massachusetts 02138 USA

**Keywords:** Optical techniques, Applied optics

*Nature Materials* **(2025)** 10.1038/s41563-025-02268-w

A recent paper by Dangyuan Lei and colleagues at the City University of Hong Kong in *Nature Materials* presents a novel quasi-4D plasmonic photothermal printing technology. This new approach overcomes long-standing limitations in micro/nano-patterning by enabling the *direct* laser patterning of functional metal-oxide (MO) materials—such as conducting ITO, semiconducting IGZO, and insulating AlO_x_—without the need for photoresists or high-temperature annealing. Unlike traditional “indirect” patterning methods (*e.g*., photolithography or nanoimprint lithography), this method uses femtosecond-laser-excited silver nanowires to generate rapid (<0.3 s) localized heating, converting MO precursors into high-performance thin films with simultaneous patterning at room temperature and in ambient air.

Critically, this single-step, additive process supports heterogeneous integration of different MOs, paving the way for all-MO electronics. The researchers demonstrated the densest all-MO transistor array to date—at 48,400 transistors/cm^2^—and functional logic gates, all fabricated without vacuum or thermal treatments. The printed devices show electrical performance comparable to those made by conventional high-temperature or vacuum-based approaches.

This universal, high-resolution, and energy-efficient printing platform sets a new paradigm for scalable, low-cost, and environmentally friendly microfabrication of next-generation electronics, with broad applications from active-matrix displays to logic circuits.

